# Mepolizumab exerts crucial effects on glucocorticoid discontinuation in patients with eosinophilic granulomatosis with polyangiitis: a retrospective study of 27 cases at a single center in Japan

**DOI:** 10.1186/s13075-023-03097-5

**Published:** 2023-06-26

**Authors:** Takashi Yamane, Akira Hashiramoto

**Affiliations:** 1 Department of Rheumatology, Kakogawa Central City Hospital, 439, Honnmachi, Kakogawa-Cho, Kakogawa, Hyogo 675-8611 Japan; 2 Department of Biophysics, Kobe University Graduate School of Health Sciences, Kobe, 654-0142 Japan

**Keywords:** Eosinophilic granulomatosis with polyangiitis, Mepolizumab, Glucocorticoid, Discontinuation

## Abstract

**Objectives:**

To investigate the efficacy of mepolizumab in patients with eosinophilic granulomatosis with polyangiitis (EGPA) and factors contributing to glucocorticoid (GC) discontinuation.

**Methods:**

We retrospectively studied EGPA patients treated with mepolizumab who were on GC at the time of induction of mepolizumab, at Japanese single center as of January 2023.

Patients were classified into those who were able to discontinue GC at the time of the investigation (GC-free group) and those who continued (GC-continue group). Patient characteristics at the time of EGPA diagnosis (age, gender, absolute eosinophil counts, serum CRP level, serum IgE level, Rheumatoid factor (RF) / anti-neutrophil cytoplasmic antibody (ANCA) positivity, presence of asthma, affected organ, Five factor score (FFS), Birmingham Vasculitis Activity Score (BVAS) and characteristics at the time of mepolizumab induction (daily prednisolone dose, concomitant immunosuppressive maintenance therapy at the mepolizumab induction, prior history of GC pulse therapy, concomitant immunosuppressive therapy for remission induction,), history of relapse before mepolizumab induction and the duration of mepolizumab treatment were compared.

We also followed the clinical indicators (absolute eosinophil counts, CRP and IgE levels, BVAS, Vascular Damage Index (VDI)) and daily prednisolone dosage at the EGPA diagnosis, at the mepolizumab induction and at the survey.

**Results:**

Twenty-seven patients were included in the study. At the time of the study, patients had received mepolizumab for median 31 months (IQR, 26 to 40), the daily prednisolone dose was median 1 mg (IQR, 0 to 1.8) and GC-free was achieved in 13 patients (48%). Among clinical indicators that have improved by conventional therapy before the induction of mepolizumab, eosinophil counts, GC doses and BVAS have successively shown significant reductions throughout the observation period both GC-free and GC-continue. Of the GC-free patients, 7 were ANCA positive and 12 had FFS1 or more. Univariate analysis showed that the absolute eosinophil counts at diagnosis was significantly higher in the GC-free group (median 8165/µl (IQR, 5138 to 13,409) vs. 4360/µl (IQR, 151 to 8380), *P* = 0.037) and significantly fewer patients presented with gastrointestinal lesions (2 (15%) vs. 8 (57%), *P* = 0.025), while multivariate analysis showed no significant differences. Mepolizumab treatment significantly improved VDI in the GC-continue group (*P* = 0.004).

**Conclusions:**

After three years of treatment with mepolizumab, approximately 50% of patients with EGPA achieved GC-free status. GC could be discontinued even in severe cases and ANCA-positive cases. Although multivariate analysis did not extract any significant factors contributing to achieving GC-free, we found that improvement in eosinophil counts and BVAS led to GC reduction, resulted in protection of organ damages in both the GC-free and continuation groups. The significance of achieving GC-free remission in EGPA patients was demonstrated.

## Introduction

Eosinophilic granulomatosis with polyangiitis (EGPA) was defined as a type of anti-neutrophil cytoplasmic antibody (ANCA)-associated vasculitis at the 2012 Revised International Chapel Hill Consensus Conference. Histologically, EGPA is characterized by eosinophilic tissue infiltration and vasculitis. EGPA presents with a variety of clinical symptoms include asthma or allergic manifestations associated with marked eosinophilia, as well as organ damage associated with vasculitis, while only about 40% of patients produce the disease-labeled antibody myeloperoxidase (MPO)-ANCA [1]. Various immunosuppressive agents, including glucocorticoid (GC), are used during each phase of remission induction and maintenance therapy [2]. In particular, relapses of disease under maintenance therapy are strongly influenced by GC dose reduction, so that almost all patients require lifelong GC administration. In a prospective study examined 115 EGPA patients who achieved remission, 41% of those relapsed within approximately 1 year after remission-induction, with 57% of these relapses occurring after the GC dose was reduced to 10 mg/day or less [3]. The GC dependence causes a variety of adverse events. Among them, infections are the second most common cause of death, especially in severe cases with increasing GC doses [4]. Mepolizumab, an anti-interleukin (IL)-5 monoclonal antibody, binds to IL-5 and prevents the interaction with its receptor on the eosinophil surface [5]. In Japan, it was approved for the treatment of resistant EGPA at a dose of 300 mg/month in 2018. Although there are established evidence of mepolizumab in prolonging the duration of remission and reducing GC [6, 7], still lacks a detailed consensus on the solution for treatment-resistant or on the pros and cons of GC discontinuation.

In this report, we retrospectively investigated the characteristics of patients with EGPA who achieved GC-free remission with mepolizumab and examined the feasibility of GC discontinuation based on real-world data from a single center in Japan.

## Methods

By using electronic medical record data, we identified EGPA patients receiving mepolizumab who were on outpatient in Kakogawa Central City Hospital as of January 2023. The Kakogawa Central City Hospital is a core hospital of the region and covers a population of approximately half a million with approximately 2000 annual outpatients visits in the rheumatology department. We classified EGPA according to the 1990 American College of Rheumatology criteria [8]. The patient background (age, gender, absolute eosinophil counts, CRP level, IgE level, Rheumatoid factor (RF) /ANCA positivity, presence of asthma, affected organ, five factor score (FFS) [9], Birmingham Vasculitis Activity Score (BVAS) [10] at diagnosis was examined. Then patients were classified into two groups (GC-free group and GC-continue group) according to the presence or absence of GC administration at the time of the study.

Patient background at the time of mepolizumab induction (daily prednisolone dose, concomitant immunosuppressive therapy for remission induction, prior history of GC pulse therapy, concomitant immunosuppressive maintenance therapy when initiating mepolizumab), history of relapse before mepolizumab induction and the duration of mepolizumab administration were examined by groups. We also examined the clinical indicators (absolute eosinophil counts, CRP level and IgE level, BVAS, Vascular Damage Index (VDI) [10]) and daily prednisolone dosage at the time of diagnosis of EGPA, at the time of induction of mepolizumab, and at the time of the investigation in January 2023. EGPA relapse was defined as the new appearance or recurrence or worsening of clinical EGPA manifestation(s) (excluding asthma and/or ear, nose, and throat (ENT)), requiring the addition or dose increase of glucocorticoids and/or other immunosuppressants [11].

The patients’ characteristics are described as median and interquartile range (IQR) for continuous variables and number and percentage (%) for categorical variables. Difference between the groups was compared using the Mann–Whitney U test, Pearson’s chi-squared test or Fisher's exact test. The Wilcoxon signed-rank test for paired samples was used for the comparison of data trends. The multivariate analyses were used to create the logistic regression models to assess the association between the independent variables and the dependent variables. The independent variables with statistically significant values (*P* < 0.05) in the univariate analysis were entered into the multivariate model by forced entry method. Odds ratios (ORs) with 95% confidence intervals (CIs) for each factor were calculated in the logistic regression. Model diagnostics were also reported by the Hosmer–Lemeshow goodness-of-fit test. The level of significance was set at *P* < 0.05. Statistical analyses were performed by SPSS version 26 (IBM Corp, Armonk, NY, USA).

## Results

Mepolizumab was administered to 30 patients with EGPA, and 27 patients who were receiving GC at the time of mepolizumab induction were examined. When mepolizumab was inducted, the median daily prednisolone dose was 5 mg, and 18 patients (67%) were on concomitant immunosuppressive agents. 10 cases were ANCA positive status (One case was pathologically diagnosed PR3-ANCA positive EGPA). The median peripheral blood eosinophil count was 431/μl, and the CRP level was 0.06 mg/dl. 8 patients (28%) had a history of relapse (Table 1).

**Table 1:**
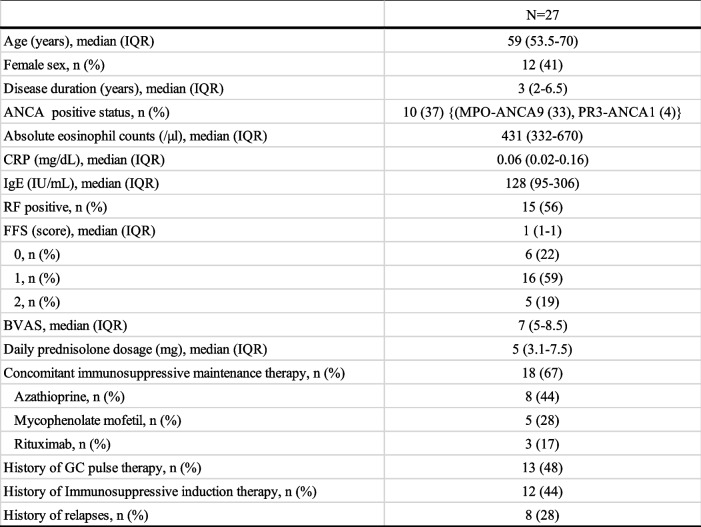
Patient background at the time of mepolizumab induction

*ANCA* anti-neutrophil cytoplasmic antibody, *RF* rheumatoid factor, *FFS* Five factor score, *BVAS* Birmingham Vasculitis Activity Score, *GC* glucocorticoid

**Fig. 1:**
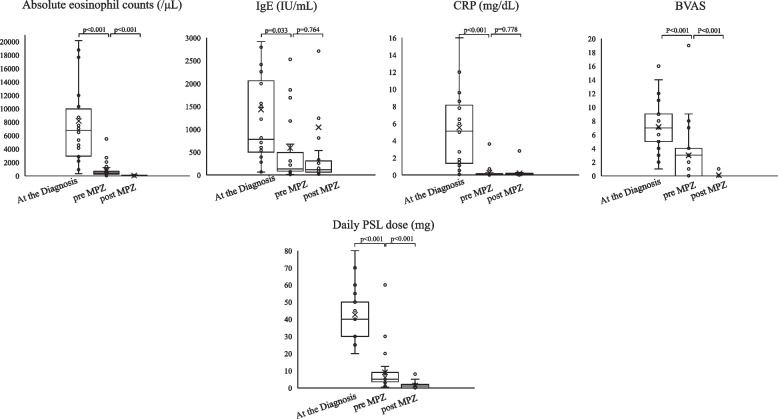
Clinical indicators and GC dose at diagnosis of EGPA and pre and post mepolizumab initiation. P values were determined by Wilcoxon signed-rank test. *P* < 0.05: baseline (at the diagnosis) vs. pre-MPZ, pre-MPZ vs. post-MPZ. *MPZ* mepolizumab, *BVAS* Birmingham Vasculitis Activity Score, *PSL* prednisolone

**Fig. 2:**
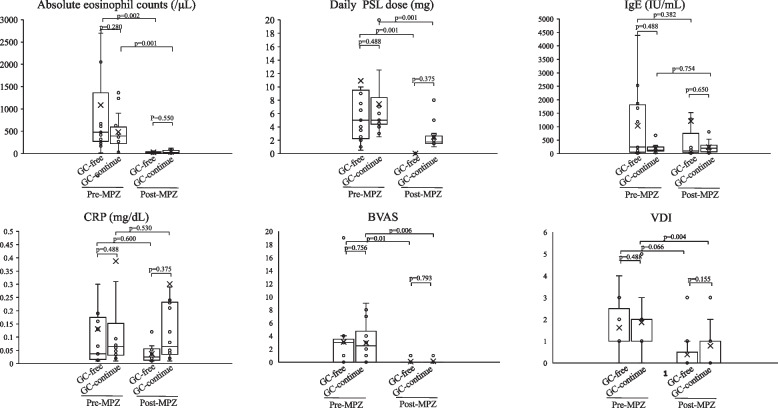
Changes in clinical indicators pre and post Mepolizumab initiation in GC-free and GC-continue patients. P values were determined by Wilcoxon signed-rank test. *P* < 0.05: pre-MPZ vs post-MPZ, GC-free vs. GC-continue at each indicator. *MPZ* mepolizumab, *PSL* prednisolone, *BVAS* Birmingham Vasculitis Activity Score, *VDI* Vascular Damage Index

Of the 27 patients, 13 (48%) achieved GC-free status. The median time from mepolizumab introduction to analysis was 31 months (IQR, 26 to 40) in the GC-free group and 39 months (IQR, 13 to 51) in the GC-continue group (*P* = 0.756). The median time from mepolizumab introduction to achieving GC-free was 9 months (IQR, 8 to 19). 7 (54%) of the GC-free cases were MPO-ANCA positive and 12 (92%) were FFS 1 or more. In univariate analysis, GC-free patients had significantly higher peripheral eosinophil counts (median 8165/μl (IQR, 5138 to 13,409) vs. 4360/μl (IQR, 151 to 8380), *P* = 0.037) and significantly fewer gastrointestinal lesions (2 cases (15%) vs. 8 cases (57%), *P* = 0.025) at the EGPA diagnosis (Table 2). A binary logistic regression analysis was performed with the absolute eosinophil count and gastrointestinal involvement (0, “not involved”, 1, “involved”), for which univariate analysis results were *P* < 0.05, as independent variables and GC-free achievement (0, “GC continue”, 1, “GC free”) as dependent variable. The logistic regression model was statistically significant, χ2(2) = 15.637, *p* < 0.001. The model explained 64.0% (Nagelkerke R2) of the variance in GC free. The Hosmer–Lemeshow test result was *p* = 0.190, and the and discriminant accuracy rate was 83.3%, indicating a good fit of the model. Results, peripheral blood eosinophil counts at the diagnosis (OR, 1.0; 95% CI, 1.00–1.001;* P* = 0.053), gastrointestinal involvement (OR, 0.001; 0.000–1.172; *P* = 0.055) may have affected on GC-free, although not significant, but the odds ratios suggest that their practical effect is low (Table 3). On the other hand, there was no difference in patient background at the time of mepolizumab induction between GC-free and GC-continue (Table 4). Prior treatment with GC and immunosuppressive agents had significantly improved peripheral blood eosinophil count, IgE and CRP, BVAS before the introduction of mepolizumab, and significantly reduced the daily GC dose. Of these, the peripheral blood eosinophil counts and BVAS showed further improvement with mepolizumab treatment (Fig. 1). When each group was examined, the introduction of mepolizumab significantly reduced the peripheral blood eosinophil counts and the daily GC dose. CRP was sufficiently reduced by prior treatments before the introduction of mepolizumab, and IgE showed a decreasing trend throughout the course of treatment. There was also no significant difference in BVAS and VDI of each group before mepolizumab. However, BVAS significantly improved in the GC-free group and VDI in the GC-continue group (Fig. 2). We had one case of relapse after mepolizumab induction who required a short course of additional GC, but remission was subsequently achieved with continued mepolizumab treatment.

**Table 2:**
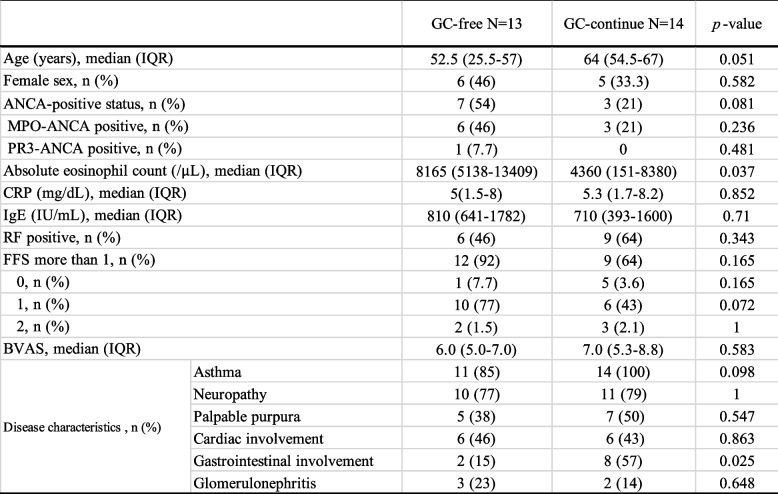
Univariate analysis of patient background at diagnosis of EGPA in patients who achieved GC-free or not

*P* values were determined by Pearson’s chi-squared test or Fisher’s exact test or Mann-Whitney U test for univariate analysis

*GC* glucocorticoid, *ANCA* anti-neutrophil cytoplasmic antibody, *RF* rheumatoid factor, *FFS* Five factor score, *BVAS* Birmingham Vasculitis Activity Score. *P* < 0.05: GC-free (*n* = 13) vs. GC-continue (*n* = 14)

**Table 3:**
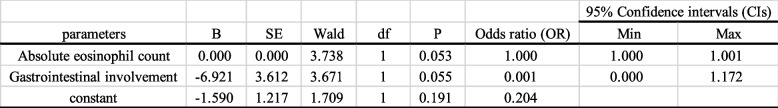
A binary logistic regression analysis with GC-free achievement as the dependent variable and univariate significant independent variables

GC free (0, “GC continue”, 1, “GC free”); Gastrointestinal involvement (0, “not involved”, 1, “involved”). *SE* standard error, *df* degrees of freedom

**Table 4:**
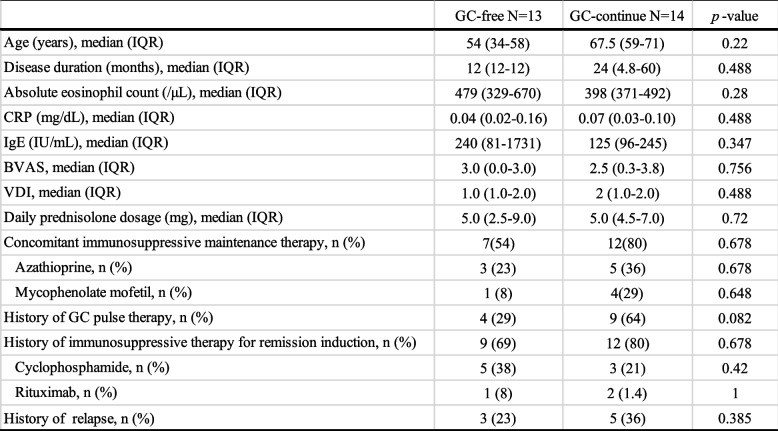
Comparison of patient background at the induction of mepolizumab in patients who achieved GC-free or not

*P* values were determined by Pearson’s chi-squared test or Fisher’s exact test or Mann-Whitney U test for univariate analysis

*BVAS* Birmingham Vasculitis Activity Score, *VDI* vasculitis damage index, *GC* glucocorticoid. *P* < 0.05: GC-free (*n* = 13) vs. GC-continue (*n* = 14)

## Discussion

This real-world trial, a median treatment of 31 months with mepolizumab, achieved GC-free status in 48% of patients including severe cases; 12 patients (92%) with FFS1 or higher and 6 patients (46%) with cardiac involvement.

In the phase 3 study of 68 patients with relapsed or refractory EGPA, GC discontinuation rate was 18% excluding those with severe disease [6]. In another study of combination therapy with mepolizumab and GC for severe EGPA, 57.1% of patients achieved a daily prednisolone dose of 5 mg or less at 6 months of treatment [12].

As in the Phase III study, the GC discontinuation rate after 48 weeks of mepolizumab treatment was 33% (9 patients) in our study. Compared with previous reports, the mean duration of mepolizumab treatment was longer in the present study. This may have contributed to the achievement of GC-free status even in severe cases, and the real-world clinical penetration of GC reduction with mepolizumab may have influenced the earlier achievement of GC-free status.

In our study, mepolizumab caused significant eosinophilia reduction in both GC-free and GC-continue groups, with associated improvement in BVAS and GC reduction. As Tsurikisawa et al. reported that GC or immunosuppressive drugs could be reduced in 20patients (66.7%) of EGPA with higher eosinophil counts at diagnosis [13], patients with marked eosinophilia are likely to have a better outcome because the condition is more likely to demonstrate the effect of IL-5 inhibition, the mechanism of action of mepolizumab. However, in multivariate analysis presenting here, eosinophilia was not a significant predictive variable for GC-free.

On the other hand, inhibition of IL-5 has influenced the pathogenesis of EGPA by suppressing not only the proliferation but also the activation of eosinophils [6]. In fact, eosinophil counts at the time of mepolizumab induction were in the normal range and no significant difference between patients with and without subsequent GC-free status in our observations. Thus, results from the phase 3 study mentioned above that 21% of EGPA patients with blood eosinophil counts below 150/μl achieved remission with mepolizumab [6], were supportive to our observations. Accordingly, an increased eosinophil count may not be a major clinical problem as a factor in achieving GC-free status, as shown by the multivariate analysis in Tables 2 and 3. In our study, conventional remission induction therapy significantly improved CRP and IgE as well as eosinophil counts at the time of mepolizumab induction. No pre-treatment drugs, GC or immunosuppressive agents, clearly contributed to these therapeutic effects. Since CRP and IgE did not show any significant decrease after mepolizumab induction, it is assumed that GC dose reduction or discontinuation was mainly due to the effect of mepolizumab on eosinophils. ANCA is a useful disease-labeled antibody in the diagnosis of vasculitis. Eosinophils are known to induce B cell proliferation and antibody production so that the suppression of ANCA production from B cells by mepolizumab is a favorable pharmacological effect in the treatment of EGPA [2]. However, previous reports suggest that mepolizumab is more effective in ANCA-negative cases and less effective in ANCA-positive cases [14], and MPO-ANCA positivity is considered a risk factor for EGPA relapse [3]. In the present study, 7 of 13 (58%) GC-free patients were ANCA-positive. Although our presenting results suggest that GC-free is an achievable goal even for ANCA-positive patients in the real world, careful observations are needed on the course, including the future relapse, of those achieve GC-free. Among the various organ damage caused by EGPA, although not significant in multivariate analysis, univariate analysis identified the presence of gastrointestinal lesions as a factor contributing to resistance to achieving GC-free. Several previous reports have also shown that EGPA with gastrointestinal lesions has a high recurrence rate and a low survival rate [15, 16]. As an additional consideration from our study, the presence of gastrointestinal lesions may have prevented the GC-free status presumably due to prolonging the duration of GC reduction.

On the other hand, VDI related to the gastrointestinal tract did not occur in this study, suggesting that careful GC dose reduction is important in diseases reported to be at high risk. Because this is a single-center, retrospective study, patient selection was limited. For the same reason, we were not able to analyze the effect of concomitant immunosuppressive agents which may have affected the clinical course. Dose reductions of GCs were not comparable to patients receiving standard therapy without mepolizumab.

## Conclusion

Although further studies are needed, our results show that approximately 3 years of mepolizumab treatment may allow GC discontinuation in about 50% of EGPA patients, even in severe cases or ANCA positive cases. In the present study of a small number of patients, no significant factors were extracted that predicted the achievement of GC-free status, but similarly, no factors that prevented GC-free status were also extracted. However, it was suggested that a slower GC dose reduction could result in a good improvement in BVAS during mepolizumab treatment and protection against organ damage, especially for patients with gastrointestinal tract involvement which has been identified as a risk factor for recurrence in previous reports.

## Data Availability

The datasets used and/or analyzed during the current study are available from the corresponding author on reasonable request.

## Abbreviations

EGPA: Eosinophilic granulomatosis with polyangiitis
ANCA: Anti-neutrophil cytoplasmic antibody
MPO: Myeloperoxidase
GC: Glucocorticoid
IL: Interleukin
RF: Rheumatoid factor
FFS: Five factor score
BVAS: Birmingham Vasculitis Activity Score
VDI: Vasculitis damage index
IQR: Interquartile range
